# Gene Targeting Turns Mice into Long-Distance Runners

**DOI:** 10.1371/journal.pbio.0020322

**Published:** 2004-08-24

Have you ever noticed that long-distance runners and sprinters seem specially engineered for their sports? One's built for distance, the other speed. The ability to generate quick bursts of power or sustain long periods of exertion depends primarily on your muscle fiber type ratio (muscle cells are called fibers), which depends on your genes. To this extent, elite athletes are born, not made. No matter how hard you train or how many performance-enhancing drugs you take, if you're not blessed with the muscle composition of a sprinter, you're not going to break the 100-meter world record in your lifetime. (In case you'd like to try, that's 9.78 seconds for a man and 10.49 seconds for a woman.)

Of course that doesn't prevent those at the highest levels from using the latest performance enhancer to get that extra 1% edge. But wait until trainers hear about the Marathon Mouse. A new study by Ronald Evans and colleagues provides evidence that endurance and running performance can be dramatically enhanced through genetic manipulation.

Skeletal muscles come in two basic types: type I, or slow twitch, and type II, or fast twitch. Slow-twitch fibers rely on oxidative (aerobic) metabolism and have abundant mitochondria that generate the stable, long-lasting supplies of adenosine triphosphate, or ATP, needed for long distance. (For more on muscle fiber metabolism, see synopsis titled “A Skeletal Muscle Protein That Regulates Endurance”) Fast-twitch fibers, which produce ATP through anaerobic glycolysis, generate rapid, powerful contractions but fatigue easily. Top-flight sprinters have up to 80% type II fibers while long-distance runners have up to 90% type I fibers. Coach potatoes have about the same percentage of both.[Fig pbio-0020322-g001]

**Figure pbio-0020322-g001:**
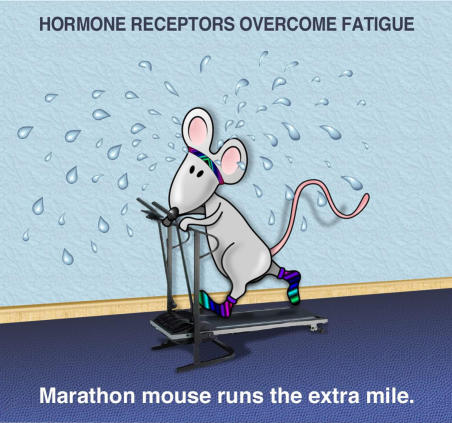


Endurance training can enhance the metabolic performance of muscle types, and now it appears that training can also induce conversion between fiber types. Specific changes in gene expression trigger this oxidative fiber transformation, but the transcription factor responsible for engineering this shift was unknown. Evans and colleagues suspected that a nuclear receptor called PPARδ—a major regulator of fat burning in fat tissue that is also prevalent in skeletal muscle—might be involved.

To investigate this possibility, the authors genetically engineered mice to express an activated form of the PPARδ protein in skeletal muscle. Type I fibers normally express higher levels of PPARδ than type II fibers, and the transgenic mice showed much higher levels of the protein than their normal littermates. The transgenic mice also had much redder muscles than the controls—type I fibers have high levels of myoglobin, the red-pigmented protein that facilitates the movement of oxygen within muscle—and elevated levels of proteins associated with mitochondrial biogenesis and operation. A final line of evidence indicating a type I fiber switch was the elevated level of specialized type I contractile proteins and decreased level of specialized type II contractile proteins in the transgenic mice. Interestingly, these same results were seen when naturally occurring (endogenous) PPARδ levels were stimulated in the normal mice (with an orally active compound). This suggests that muscle fibers can be transformed into type I endurance fibers by simply activating the endogenous PPARδ pathway.

In a weight-conscious world, oxidative fibers are thought to offer resistance against obesity since obese individuals have fewer type I fibers than average-weight individuals. Sure enough, transgenic mice fed a high-fat diet gained far less weight than normal mice fed the same diet, even in the absence of exercise. The transgenic mice had much smaller fat cells, which the authors attribute to enhanced oxidative capacity of the muscle tissue, and improved glucose tolerance. (Obese individuals lose the ability to metabolize glucose, which puts them at risk for diabetes.) But what about performance? Remarkably, the marathon mice ran about an hour longer than controls, showing dramatic improvement in both running time and distance—increases of 67% and 92%, respectively.

Altogether, these results show that PPARδ drives the conversion of type I muscle fibers by activating pathways that enhance physical performance and protect against obesity. The finding that endurance and running capacity can be genetically manipulated suggests that muscle tissue is far more adaptable than previously thought. Maybe Olympiads can be made after all—but don't give up on training just yet. A full understanding of the molecular basis of muscle fiber determination, including the interactions between PPARδ and its regulatory components, awaits further study.

